# Decreased expression of *FOXF2* as new predictor of poor prognosis in stage I non-small cell lung cancer

**DOI:** 10.18632/oncotarget.10876

**Published:** 2016-07-28

**Authors:** Peng-Zhou Kong, Guang-Ming Li, Yin Tian, Bin Song, RuYi Shi

**Affiliations:** ^1^ Translational Medicine Research Center, Shanxi Medical University, Taiyuan 030001, China; ^2^ Key Laboratory of Cellular Physiology, Ministry of Education, Shanxi Medical University, Taiyuan 030001, China; ^3^ School of Basic Medical Sciences, Tianjin Medical University, Tianjin 300070, China; ^4^ Department of General Surgery, The Second Hospital of JingZhou, JingZhou 434000, China; ^5^ Department of Biochemistry and Molecular Biology, Tianjin Medical University Cancer Institute and Hospital, Tianjin 300060, China; ^6^ Department of Oncology, The First Hospital, Shanxi Medical University, Taiyuan 030001, China; ^7^ Department of Cell Biology and Genetics, Shanxi Medical University, Taiyuan 030001, China

**Keywords:** FOXF2, non-small-cell lung carcinoma, survival, clinical stage

## Abstract

**Background:**

Forkhead box F2 (*FOXF2*) is relatively limited to the adult lung, but its contribution to non-small cell lung cancer (NSCLC) prognosis is unclear.

**Results:**

*FOXF2* mRNA levels in NSCLC were lower than that in paired normal lung tissues (*P* = 0.012). The FOXF2_low_ patients had shorter survival time than the FOXF2_high_ patients (*P* = 0.024) especially in stage I (*P* = 0.002), chemotherapy (*P* = 0.018) and < 60 age groups (*P* = 0.002). Lower *FOXF2* mRNA levels could independently predict poorer survival for patients with NSCLC (HR = 2.384, 95% CI = 1.241–4.577; *P* = 0.009), especially in stage I (HR =4.367, 95% CI =1.599–11.925; *P* = 0.004). The two independent datasets confirmed our findings.

**Methods:**

We examined *FOXF2* mRNA levels in 84 primary NSCLC and 8 normal lung tissues using qRT-PCR. Rank-sum tests and chi-square tests were used to assess the differences among groups with various clinicopathological factors. Kaplan-Meier tests were used to compare survival status in patients with different *FOXF2* mRNA levels. Cox proportional hazards regression model was used to evaluate the predictive value of *FOXF2* mRNA level in NSCLC patients. Independent validation was performed using an independent dataset (98 samples) and an online survival analysis software Kaplan-Meier plotter (1928 samples).

**Conclusions:**

Our results demonstrated that decreased *FOXF2* expression is an independent predictive factor for poor prognosis of patients with NSCLC, especially in stage I NSCLC.

## INTRODUCTION

Lung cancer is by far the main cause of cancer-related death. It is the most frequently diagnosed cancer and the leading cause of cancer death in males and the second in females [1, 2]. Lung cancer is usually classified into two main types: small cell lung cancer (SCLC) and non-small cell lung cancer (NSCLC) depending upon the microscopic appearance of the tumor cells. NSCLC is the most common type of lung cancer, accounting for 85% of all lung cancers diagnosed [3]. Currently, for NSCLC patients, the most accurate prognostic factors are tumor size, node, and tumor-node-metastasis (TNM) staging. However, as a heterogeneous disease, even with similar clinical and pathological features, and similar TNM stage, patients with NSCLC may have different outcomes due to distinct inherent biological characteristics of the tumor. Therefore, new prognostic factors are needed to be determined to better predict the outcome of lung cancer and provide a potential improvement in better treatment, especially within a given TNM stage.

Forkhead box F2 (*FOXF2*) is a member of Forkhead box transcription factors family, which is characterized by a highly conserved 110 amino acid DNA binding domain [4] and function as an activator or inhibitor of gene transcription [5]. Human *FOXF2* was initially identified in 1994 [6], and the gene is located at 6p25.3 [7]. *FOXF2* was found to have a relatively restricted high-expression limited to the adult lung and transactivated pulmonary surfactant proteins A, B, and C (SPA, SPB, and SPC) [8]; however, later studies revealed it had a more widespread expression [9]. FOXF2 plays an important role in embryonic development [10, 11], extracellular matrix synthesis [11] and epithelial-mesenchymal interaction [9], and the knockout of foxf2 mice present with cleft palate or a range of defects, including megacolon, colorectal muscle hypoplasia and agangliosis.

In cancer, FOXF2 has been considered as a potential tumor suppressor. In our previous studies, decreased FOXF2 expression was associated with early-onset metastasis and poor prognosis for patients with triple-negative breast cancer [12], and further studies showed that FOXF2 can inhibit epithelial-mesenchymal transition (EMT) and metastasis of basal-like breast cancer by targeting TWIST1 [13] and FOXC2 [14] directly. In prostate cancer, FOXF2 mRNA was decreased [15, 16] compared to normal prostate tissues, and it is a potential target genes of miR-182-5p, which promotes cell invasion and proliferation by down- regulating FOXF2, RECK and MTSS1 genes [17]. And in breast cancer FOXF2 is a target gene of miR-301, which acts as a crucial oncogene to promote metastatic tumor progression [18]. The evidence given above indicates FOXF2 may act as a tumor suppressor in tumorigenesis and metastasis.

However, the role of FOXF2 in lung cancer is unknown, especially in NSCLC. In this current study, our results showed that mRNA of FOXF2 was significantly decreased in NSCLC tissues compared to paired normal lung tissues. Additionally decreased FOXF2 mRNA expression was associated with poor prognosis in Stage I NSCLC patients, and it could predict poor prognosis for patients with Stage I NSCLC independently.

## RESULTS

### Expression level of *FOXF2* mRNA in lung cancer tissues

**Figure 1:**
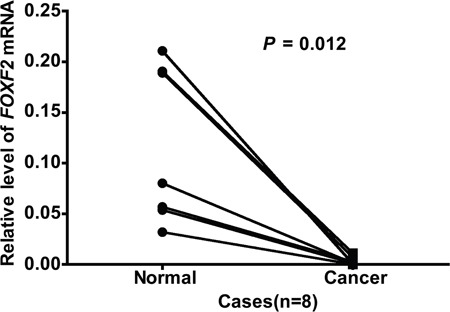
Comparison of *FOXF2 mRNA* expression in paired lung tumor tissues and normal tissues The mRNA of *FOXF2* was significantly decreased in cancer tissue compared with paired normal lung tissue in all 8 cases.

First, we measured the *FOXF2* mRNA levels in primary lung cancer and paired normal samples from patients with NSCLC using real-time PCR analysis. The mRNA level of *FOXF2* ranged from 1.79E-04 to 157.47 in primary lung cancers and the median was 5.86E-03. The mRNA level of *FOXF2* ranged from 3.20E-02 to 2.11E-01 in normal lung tissues and the median was 6.86E-02. Significant difference in *FOXF2* mRNA levels was found between paired primary lung cancers and normal lung tissues (*P* = 0.012, Z = −2.521, Figure 1). All cancer samples were grouped into two groups: *FOXF*2_low_ (≤ 3.75E-03) and *FOXF2*_high_ (> 3.75E-03), according to the ROC curve analyses (AUC=0.657, *P*=0.021, 95% confidence interval:0.531-0.782). The disease-free survival (DFS) of *FOXF*2_low_ ranged from 1 month to 54 months, and the median was 26 months. The DFS of *FOXF2*_high_ ranged from 1 month to 59 months, and the median was 46 months. Rank-sum test shown the patients in the *FOXF2*_high_ group had a longer survival time than those in the *FOXF*2_low_ group (Z = −2.347, *P* = 0.019).

### Correlation between the mRNA level of *FOXF2* and clinicopathologic factors

To establish the link between *FOXF2* mRNA levels in primary tumors and clinicopathological features of lung cancer, we analyzed the *FOXF2* mRNA levels among different clinicopathologic groups. No significant difference of *FOXF2* mRNA levels was found in patients with different gender, age, histology, clinical stage, family history, and smoking history (*P* > 0.05, Table 1). Although no significant difference was found among the three tumor size groups (*P* = 0.063, Table 1), the mRNA of *FOXF2* in the size ≤3cm group was significantly higher than in the size > 7cm group (*P* = 0.037, Table 1).

**Table 1:** Association of *FOXF2* mRNA levels with clinicopathological factors in patients with NSCLC

Variables	Cases	Median levels of *FOXF2* (1×10^−3^)	Rank sum tests		*FOXF2* mRNA level		Chi-square test	
			Z/χ^2^	*P*	Low (%)	High (%)	χ^2^	*P*
**Age (years)**								
<60	34	7.16(0.18-157477)	−1.394	0.163	10 (29.4%)	24(70.6%)	1.377	0.241
≥60	50	4.69(0.27-622.01)			21 (42.0%)	29 (58.0%)		
**Gender**								
Female	22	9.09(0.27-217.19)	−1.312	0.189	6(27.3%)	16(72.7%)	1.188	0.276
Male	62	4.87(0.18-157477)			25 (40.3%)	37(59.7%)		
**Family history**								
Yes	9	7.15(1.72-29.18)	−0.108	0.914	4 (44.4%)	5 (55.6%)	0.246	0.620
No	75	5.22(0.18-157477)			27 (36.0%)	48 (64.0%)		
**Smoking history**								
Current	68	4.96(0.18-157477)	−1.367	0.172	25 (36.8%)	43 (63.2%)	0.003	0.956
Never	16	10.61(0.51-217.19)			6 (37.5%)	10 (62.5%)		
**Tumor size (cm)**								
≤3	27	7.15 (0.51-49.65)	1.648	0.439	7 (25.9%)	20 (74.1%)	5.440	0.063
3-7	52	5.86 (0.18-157477)			20 (38.5%)	32 (61.5%)		0.265*
>7	5	2.40 (1.18-261.88)			4 (80.0%)	1 (20.0%)		0.037**
								0.151***
**Clinical stage**								
I	42	4.96 (0.64-65.56)	1.118	0.572	18 (42.9%)	24 (57.1%)	1.859	0.395
II	22	7.08 (0.27-261.88)			8 (36.4%)	14 (63.6%)		
III-IV	20	6.00 (0.18-157477)			5 (25.0%)	15(75.0%)		
**Histology type**								
Squamous cell carcinoma	62	4.87 (0.18-157477)	−1.628	0.104	25 (40.3%)	37(59.7%)	1.188	0.276
Adenocarcinoma	22	7.74(0.27-217.19)			6(27.3%)	16(72.7%)		

* ≤3 vs 3-7;

** ≤3 vs >7;

*** 3-7 vs >7.

### *FOXF2* mRNA levels reflected the DFS status in NSCLC patients

**Figure 2:**
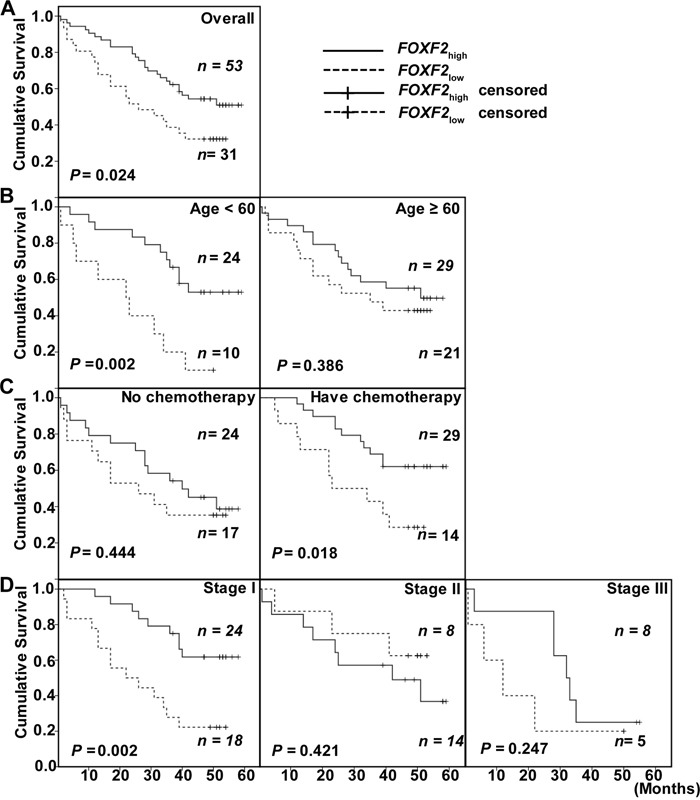
Kaplan-Meier survival curves of patients with different *FOXF2 mRNA* expression **A.** Cumulative DFS in the overall study population. **B.** Cumulative DFS of patients in the age <60 group and in the age ≥60 group. **C.** Cumulative DFS in patients accepting chemotherapy or no chemotherapy. **D.** Cumulative DFS in stage I, II, III NSCLC.

To explore the relationship between *FOXF2* mRNA levels in primary tumors and DFS status of lung cancer patients, Kaplan-Meier survival analysis was used to compare the DFS status of lung cancer patients with different *FOXF*2 mRNA expression status. In the overall study population (*n* = 84), patients with low *FOXF2* levels had a statistically lower cumulative DFS than those with high *FOXF2* levels (*P* = 0.024, Figure 2A). In different gender, age ≥60, tumor size, histology types, family, or smoking history groups, there was no difference in disease-free survival time between *FOXF*2_low_ and *FOXF*2_high_ patients. In the age <60 group, the patients with low *FOXF2* levels had a statistically lower cumulative DFS than those with high *FOXF2* levels (*P* = 0.002, Figure 2B). When receiving chemotherapy, the patients with low *FOXF2* levels had a statistically shorter cumulative DFS than those with high *FOXF2* levels (*P* = 0.018, Figure 2C). Although there was no difference in stage II and III groups, in the stage I group *FOXF2* expression significantly affected the survival time of lung cancer patients and the *FOXF2*_low_ group had a significantly lower survival time than the *FOXF2*_high_ group (*P* =0.002, Figure 2D). And the 7 patients in stage IV all belong to FOXF2_low_ group and didn't perform Kaplan-Meier analysis and Log-rank test.

### Prediction of disease-free survival based on the mRNA level of *FOXF2*

To evaluate the predictive value of *FOXF2* mRNA level for DFS status in lung cancer patients, *FOXF2* mRNA and other factors were used to make the univariate analysis by cox proportional hazard regression model. Univariate analysis showed that *FOXF2*_low_ and tumor size were significant risk factors in predicting DFS status in the overall study population (*FOXF2*, hazard ratio (HR) = 1.927, 95% CI = 1.077–3.449, *P* = 0.027; ≤3 vs. >7, HR = 0.308, 95% CI = 0.111–0.854, *P* = 0.024; 3-7 vs. >7, HR = 0.256, 95% CI = 0.097–0.672, *P* = 0.006) and in stage I NSCLC patients (*FOXF2*, HR = 3.526, 95% CI = 1.510–8.231; *P* = 0.004; ≤3 vs. >7, HR = 0.345, 95% CI = 0.110–1.080, *P* = 0.067; 3-7 vs. >7, HR = 0.222, 95% CI = 0.075–0.662, *P* = 0.007) (Table 2).

**Table 2:** Univariate and multivariate Cox models for the association between survival and clinicopathological factors in patients with NSCLC

Variables		Univariate analysis			Multivariate analysis		
		HR	95% CI	*P-*value	HR	95% CI	*P-*value
**Age**	**<60 vs. ≥60**	1.127	0.629-2.021	0.688	1.194	0.612-2.331	0.603
**Gender**	**Female vs Male**	0.472	0.220-1.013	0.054	0.520	0.214-1.263	0.149
**Histology**	**Sq. vs. Ade.**	1.094	0.566–2.113	0.789	0.840	0.382-1.850	0.666
**Smoking history**	**no vs yes**	0.679	0.303-1.518	0.345	0.563	0.197-1.612	0.285
**Family history**	**no vs yes**	0.761	0.300-1.927	0.564	0.864	0.323-2.314	0.772
**Tumor size (cm)**	**≤3 vs. >7** **3-7 vs. >7**	0.308 0.256	0.111–0.854 0.097-0.672	0.024 0.006	0.345 0.222	0.110-1.080 0.075-0.662	0.067 0.007
**Clinical stage**	**II vs. I** **III-IV vs. I**	0.898 1.277	0.437–1.843 0.635-2.571	0.769 0.492	0.674 1.862	0.310-1.465 0.859-4.035	0.320 0.115
***FOXF2* mRNA**	**Low vs. High**	1.927	1.077–3.449	0.027	2.384	1.241-4.577	0.009

Sq. means Squamous cell carcinoma.

Ade. means Adenocarcinoma

**Figure 3:**
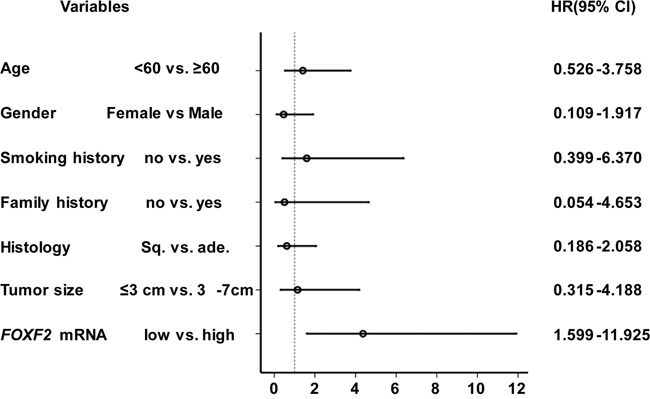
Predictive value of *FOXF2* mRNA level in primary cancer tissues for prognosis of patients with stage I NSCLC Multivariate analysis by cox proportional hazards regression model showed *FOXF2* mRNA level was an independent prediction factor for survival of patients with stage I NSCLC.

Furthermore, multivariate analysis was carried out to evaluate the *FOXF2* mRNA and other significant factors with a cox proportional hazard regression model. The result showed that in patients with NSCLC, *FOXF2* mRNA level was an independent prediction factor for survival and the *FOXF2*_low_ patients had a shorter survival time than the *FOXF2*_high_ patients (HR = 2.384, 95% CI = 1.241–4.577; *P* = 0.009) especially in stage I NSCLC patients (HR = 4.367, 95% CI =1.599–11.925; *P* = 0.004) (Table 3, Figure 3).

**Table 3:** Univariate and multivariate Cox models for the association between survival and clinicopathological factors in patients with stage I NSCLC

Variables		Univariate analysis			Multivariate analysis		
		HR	95% CI	*P-*value	HR	95% CI	*P-*value
**Age**	**<60 vs. ≥60**	1.350	0.570-3.196	0.494	1.407	0.526-3.758	0.496
**Gender**	**Female vs Male**	0.535	0.182-1.575	0.256	0.458	0.109-1.917	0.285
**Histology**	**Sq. vs. ade.**	0.710	0.300–1.678	0.435	0.619	0.186-2.058	0.434
**Smoking history**	**no vs. yes**	1.375	0.542-3.493	0.503	1.594	0.399-6.370	0.510
**Family history**	**no vs. yes**	0.869	0.117-6.464	0.891	0.503	0.054-4.653	0.545
**Tumor size (cm)**	**≤3 vs. 3-7**	1.621	0.601–4.371	0.340	1.149	0.315-4.188	0.834
***FOXF2* mRNA**	**Low vs. High**	3.526	1.510–8.231	0.004	4.367	1.599-11.925	0.004

Sq. means Squamous cell carcinoma.

Ade. means Adenocarcinoma

### Independent validation

**Figure 4:**
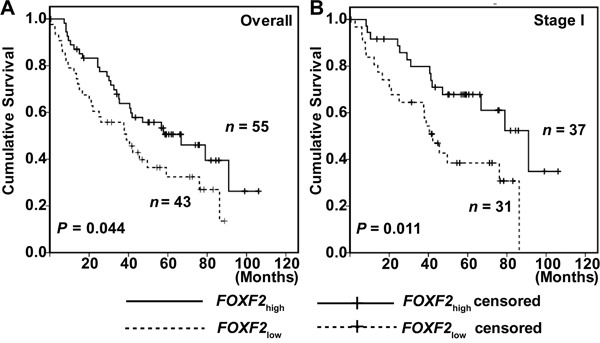
Kaplan-Meier survival curves of patients with different *FOXF2 mRNA* expression of the independent validation **A.** Cumulative DFS in overall independent validation population. **B.** Cumulative DFS in stage I group of the validation.

The prognostic value of expression of *FOXF2* mRNA was validated in an independent dataset consisting of 17 normal lung tissues and 98 lung cancer tissues from the study of Bhattacharjee [19]. In these validation samples, the median mRNA level of FOXF2 in primary lung cancers was lower than that in normal lung tissues (median level, -1.450945 vs 0.16391; *P* = 2.40E-5). NSCLC patients with *FOXF2*_low_ had a poorer DFS (median months, 37.6 vs 47.2; *P* = 0.065) than *FOXF2*_high_ patients. Kaplan-Meier survival analysis showed that *FOXF2*_low_ patients had a poorer DFS than *FOXF2*_high_ patients in the overall study population (*P* = 0.044; Figure 4A) and in the stage I group (*P* = 0.011, Figure 4B). The multivariate analysis showed that *FOXF2* mRNA level was an independent prediction factor for overall survival (HR =1.880, 95 % CI: 1.082–3.268, *P* = 0.025) and in stage I group (HR = 2.278, 95 % CI: 1.106-4.690, *P* = 0.025) (see Supplementary Table S1 and S2).

Another validation was performed using an online survival analysis software, Kaplan-Meier plotter, consisting of 2,437 lung cancer patients with a mean follow-up of 49 months [20]. In the validation samples (n = 1928), Kaplan-Meier survival analysis showed that NSCLC patients with *FOXF2*_low_ had a poorer 5-year DFS than *FOXF2*_high_ patients in the overall population (HR = 0.6 (0.53 − 0.7), log-rank *P =* 1.8e−12, Figure S1A) and in the stage I group (HR = 0.47 (0.35 − 0.65), log-rank *P =* 1.2e−06, Figure S1B). There was no difference between *FOXF*2_low_ and *FOXF*2_high_ patients in the stage II group (HR = 1.09 (0.74 – 1.61), log-rank *P =* 0.66, Figure S1C) or in the stage III group (HR = 0.67 (0.38 − 1.21), log-rank *P =* 0.19, Figure S1D). Patients with *FOXF2*_low_ had a poorer DFS than *FOXF2*_high_ patients in the lung adenocarcinoma population (HR = 0.4 (0.29 − 0.54), log-rank *P =* 1.6e-09, Figure S2A), in the lung squamous cell carcinoma population (HR = 0.66 (0.49 − 0.89), log-rank *P =* 0.0057, Figure S2B), and in the never smoked group (HR = 0.39 (0.2 − 0.74), log-rank *P =* 0.0027, Figure S2C) and in the smoked group respectively (HR = 0.54 (0.41 − 0.69), log-rank *P* = 1.8e−06, Figure S2D).

## DISCUSSION

Forkhead proteins are a large family of transcription factors and are commonly associated with development. Recent studies have shown that the FOX transcription factors play important roles in tumor progression in several types of cancers [21–32]. In our present study, the mRNA level of *FOXF2* was found to be decreased in primary lung cancer compared with paired normal lung tissue, and it negatively correlated with the size of lung cancer. Furthermore, low expression of *FOXF2* was associated with the worst outcome of NSCLC patients with clinical stage I. And two independent validation studies (n = 98 and n = 1928 respectively) confirmed our findings. So we suppose FOXF2 is a predictor of NSCLC prognosis.

It has been reported that FOXF2 plays an important role in epithelial-mesenchymal interactions [9] and inhibition of Foxf2 leads to loss of collagen synthesis [11]. This indicates FOXF2 is an important regulator in extracellular matrix (ECM) production and remodeling [11]. FOXF2 modulates ECM balance and remodeling through regulating the balance between MMPs and TIMPs [15, 16]. In prostate cancer, MMP1 was down-regulated by FOXF2 whereas TIMP3, one of MMPs inhibitors, was up-regulated by FOXF2. Additionally, in prostate cancer, FOXF2 has an opposite regulatory effect with TGFβ3 pathways [15, 16], which is described as triggering EMT via MMP-dependent mechanisms [33, 34]. In our previous study, we found that FOXF2 is a novel EMT-suppressor [13] and decreased FOXF2 is associated with poor prognosis of patients with basal-like breast cancer [12]. Our current results show FOXF2 levels were decreased in lung cancer tissue and its down-regulation is associated with the increased tumor size. It is possible that the decreased FOXF2 leads to an imbalance in matrix synthesis/degradation and provides a suitable environment for the growth and metastasis of cancer cells and leads to a worse outcome at last. So FOXF2 might be a tumor suppressor and work by maintaining the balance of ECM and inhibition of EMT. However, recent studies showed Foxf2 induced robust EMT, migration, invasion and metastasis in lung cancer cells [35], and inhibition of miR301 enhances Akt-mediated cell proliferation and FoxF2 is a regulatory target for miR301 [36]. Thus, further investigations are required to identify the role of FOXF2 in lung cancer and other cancer types.

The most common cause of lung cancer is long-term exposure to tobacco smoke [37, 38]. Tharappel et al. have shown that cigarette smoke exposure leads to quantitative increases in DNA binding activities of Foxf2 after only 10 days in mice [39]. In the present study, although there is no statistical significance, the expression of *FOXF2* mRNA in the non-smoking group is higher than in the smoking group. However, the effect of smoke on *FOXF2* in lung cancer needs to be investigated furtherly.

Another interesting thing is that in age < 60 group decreased *FOXF2* mRNA levels may be a marker of lower survival of NSCLC. It has been reported that age is an important predictor of prognosis in lung cancer patients [40–42] and older patients have a worse outcome compared with younger patients. However, in younger patients with the same TNM stage, tumors may have different metastatic potential or even form a different metastatic phenotype and lead to a different prognosis. Our investigation revealed that in age < 60 group, patients with decreased *FOXF2* mRNA levels had a lower survival rate than patients with the high *FOXF2* mRNA level. *FOXF2* mRNA levels might be a potential molecular predictor of prognosis in young patients with NSCLC.

In conclusion, our results demonstrate the prognostic value of *FOXF2* mRNA expression in patients with NSCLC. *FOXF2* mRNA expression negatively correlates with the size of NSCLC, and patients with high-expressing *FOXF2* mRNA have significantly better survival than patients with low-expressing *FOXF2*. FOXF2 may inhibit growth and metastasis of cancer cells by regulating ECM remodeling and EMT process or other mechanisms. Decreased *FOXF2* is a promising candidate for predicting poor prognosis in Stage I NSCLC.

## MATERIALS AND METHODS

### Clinical samples

All 92 lung tissues, including 84 lung cancer and 8 paired normal lung tissues, were collected from lung cancer patients (age range: 40-79; mean age: 62) without preoperative chemotherapy. Patients underwent complete resection of cancer followed by radiotherapy (6 cases), paclitaxel plus cisplatin chemotherapy (43 cases), combined radiation and chemotherapy (13 cases), or supportive care only from June 1995 to January 2005 at Tianjin Cancer Hospital, China. Of these cases, primary cancers and paired normal lung tissues were collected in 8 cases, and only primary cancer samples were obtained in the other 76 cases. Tissue samples were diagnosed as NSCLC using hematoxylin and eosin (H&E) staining, and only samples with 75% or more tumor cells in primary tumors were selected for quantitative real-time RT-PCR. Clinical staging of cancer was determined according to American Joint Commission for Cancer (AJCC)/International Union Against Cancer (UICC) TNM staging system and 42, 22, 13, and 7 patients present with stage I, II, III, and IV, respectively. The use of these tissues was approved by the Institutional Reviewing Board and the Research Committee, and written consent was obtained from all participants. Disease-free survival (DFS) time was defined as the time from primary surgery to any relapse (local-regional, contra-lateral and/or distant), death or terminal time of follow-up without any relapse events. Another dataset including 17 normal lung tissues and 98 lung cancer tissues from patients with complete follow-up data according to the study of Bhattacharjee [19] acts as an independent validation. A large cohort including 2,437 samples of ten independent datasets was used by the online survival analysis software [20].

### RNA extraction and cDNA preparation

Tissue specimens from cancer were snap-frozen in liquid nitrogen within 30 minutes after dissection and then stored at −80°C. RNA was extracted with TRIZOL reagent (Life technologies, Gaithersburg, MD, USA) according to the manufacturer's instructions. Five μg of total RNA were used to perform RT for the first-strand cDNA synthesis. In brief, RNA was denatured for 5 minutes at 65°C and snap-cooled on ice in the presence of 0.5 μg Oligo (dT) and 10 mmol dNTP mix, followed by incubation at 4°C for 50 minutes with First-Strand Buffer, 0.2 μmol DTT, 40 U RNaseOUT ribonuclease inhibitor and 200 U SuperScript II in total volume 20 μL reaction system. Reactions were stopped by incubation at 70°C for 15 minutes.

### Real-time PCR

Real-time PCR analysis was performed using the Platinum® Quantitative PCR SuperMix-UDG (Life Technologies) according to the manufacturer's instructions. Primers and Taqman probes of FOXF2 and the housekeeping gene, glyceraldehyde-3-phosphate dehydrogenase (GAPDH), were used as previously described [12]. Assays were performed with the ABI 7500 TaqMan system (Applied Biosystems, Foster City, CA, USA). PCR was carried out after incubation at 50°C for 2 minutes and pre-denaturing at 95°C for 3 minutes followed by 40 cycles at 95°C for 30 s and 58°C for 1 minute. Quantitation of the expression of the target gene in samples was accomplished by measuring the fractional cycle number at which the amount of expression reaches a fixed threshold (CT). The relative quantitation was given by the CT values, determined by triplicate reactions for test and reference samples for the target gene and for GAPDH. Triplicate CT values were averaged and the GAPDH CT was subtracted to obtain ΔCT. The relative expression level of the target gene was determined to be 2^−ΔCT^.

### Statistical analysis

The Receiver Operating Characteristic (ROC) curve was used to identify the optimized cut-off value of *FOXF2* mRNA level which separated the participants into two groups: the *FOXF2*_high_ group and *FOXF2*_low_ group, respectively. A paired rank-sum test was used to analyze the mRNA expression differences between primary lung cancers and paired normal lung tissue. Wilcoxon rank-sum tests or Kruskal-Wallis H tests were used to compare mRNA expression differences between/among different clinicopathologic groups. Survival analysis was carried out according to Kaplan–Meier analysis and Log-rank test. Univariate and multivariate survival analyses were performed by a Cox proportional hazards regression model. All calculations were performed with SPSS for Windows statistical software package (SPSS Inc, Chicago, IL, USA). *P*-values of less than 0.05 were considered statistically significant.

## SUPPLEMENTARY FIGURES AND TABLES


