# Supplementary material for: Decreased expression of FOXF2 as new predictor of poor prognosis in stage I non-small cell lung cancer
Source: Oncotarget. 2016 Jul 28;7(34):55601–10. doi: 10.18632/oncotarget.10876 (PMC5342439; doi:10.18632/oncotarget.10876)
Supplement: Supplementary file 1 [file oncotarget-07-55601-s001.pdf]

Decreased expression of *FOXF2* as new predictor of poor prognosis in stage I non-small cell lung cancer

SUPPLEMENTARY FIGURES AND TABLES

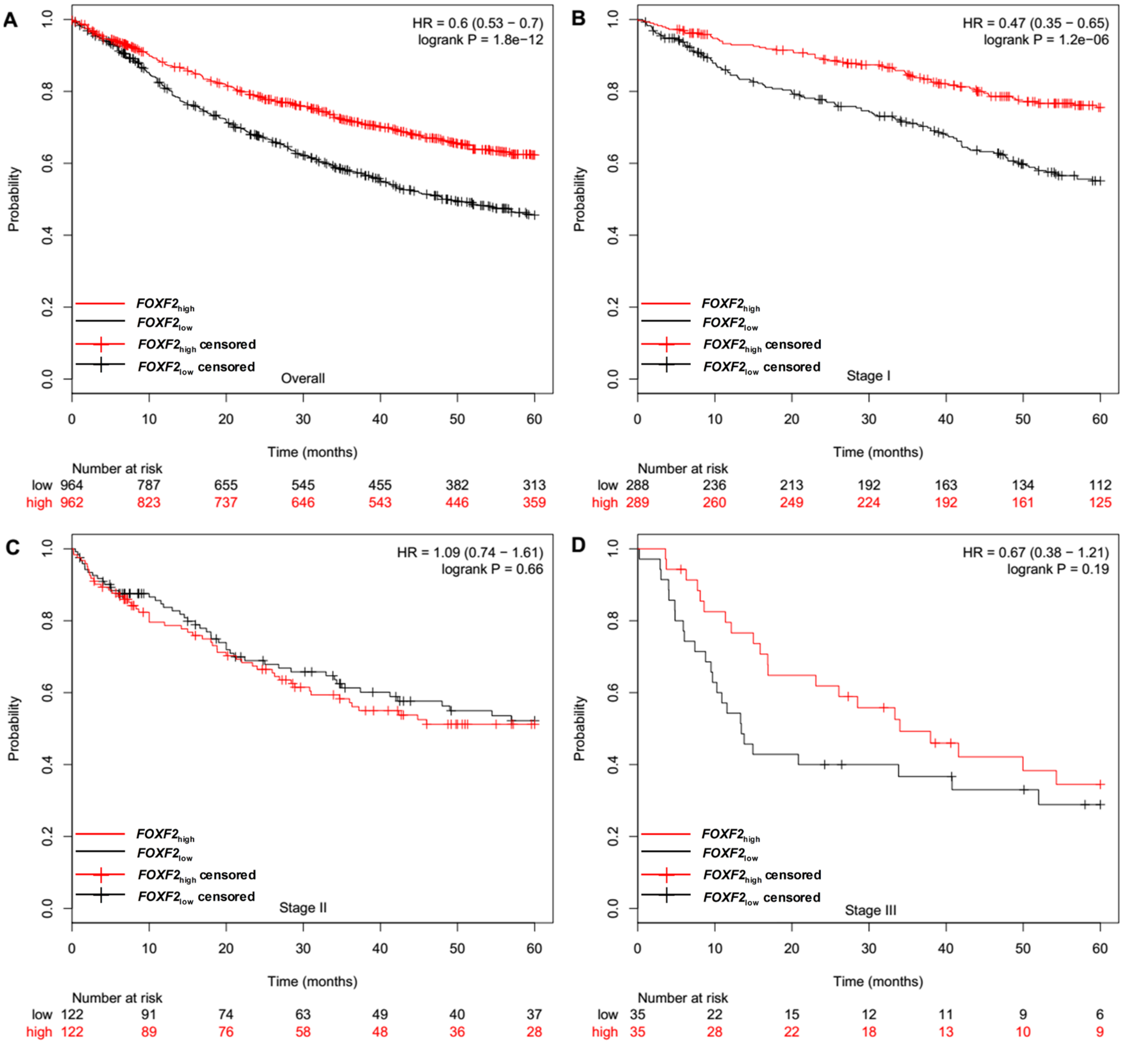

Supplementary Figure S1: Kaplan-Meier survival curves of patients with different *FOXF2 mRNA* expression using the online survival analysis software Kaplan-Meier plotter. A. Cumulative DFS in overall independent validation population. **B.** Cumulative DFS in stage I group of the validation. **C.** Cumulative DFS in stage II group of the validation. **D.** Cumulative DFS in stage III group of the validation.

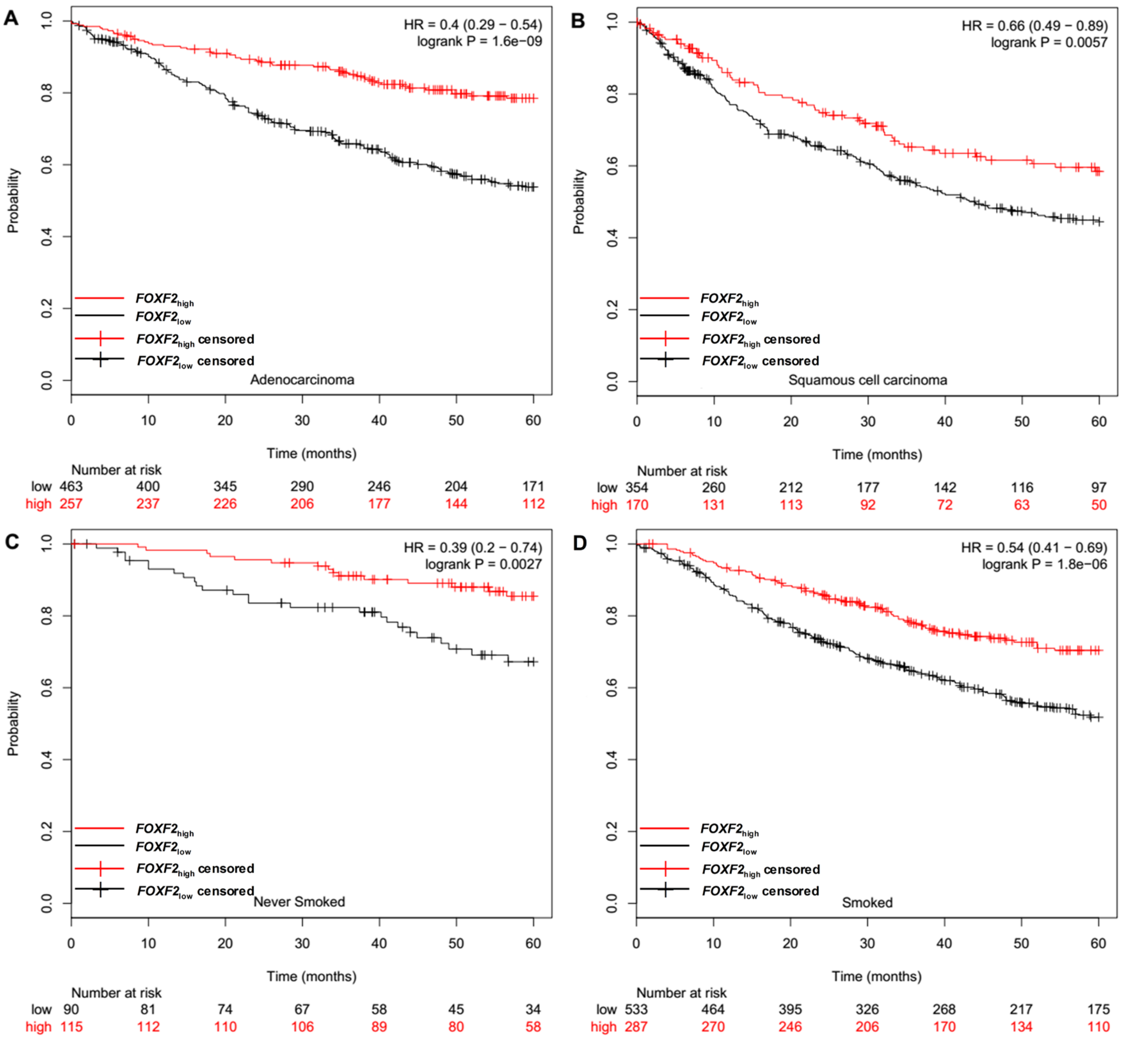

Supplementary Figure S2: Kaplan-Meier survival curves of patients with different *FOXF2 mRNA* expression using the online survival analysis software Kaplan-Meier plotter. **A.** Cumulative DFS in lung adenocarcinoma population. **B.** Cumulative DFS in lung squamous cell carcinoma population. **C.** Cumulative DFS in never smoked population. **D.** Cumulative DFS in smoked population.

Supplementary Table S1: Univariate and multivariate Cox models for the association between survival and clinicopathological factors in patients with NSCLC (validation)

Variables		Univariate analysis			Multivariate analysis		
		HR	95% CI	*P*-value	HR	95% CI	*P*-value
**Age**	**<60 vs. ≥60**	0.924	0.531-1.607	0.780	0.564	0.290-1.098	0.092
**Gender**	**Female vs Male**	0.731	0.437-1.222	0.054	0.722	0.413-1.262	0.253
**Smoking history**	**no vs yes**	1.557	0.839-2.891	0.345	1.336	0.642-2.784	0.439
**Clinical stage**	**II vs. I**	1.607	0.790–3.270	0.764	1.596	0.778-3.275	0.202
	**III-IV vs. I**	3.561	1.921-6.602	0.108	5.118	2.427-10.795	0.000
***FOXF2* mRNA**	**Low vs. High**	1.705	1.008–2.884	0.047	1.880	1.082-3.268	0.025

Supplementary Table S2: Univariate and multivariate Cox models for the association between survival and clinicopathological factors in patients with stage I NSCLC (validation)

Variables		Univariate analysis			Multivariate analysis		
		HR	95% CI	*P*-value	HR	95% CI	*P*-value
**Age**	**<60 vs. ≥60**	0.573	0.248-1.321	0.191	0.628	0.266-1.482	0.289
**Gender**	**Female vs Male**	0.735	0. 369-1.463	0.381	0.830	0.410-1.678	0.604
**Smoking history**	**no vs. yes**	1.477	0.563-3.873	0.428	1.406	0.522-3.785	0.501
***FOXF2* mRNA**	**Low vs. High**	2.418	1.196–4.881	0.014	2.278	1.106-4.690	0.025
